# Rapamycin Combined with α-Cyanoacrylate Contributes to Inhibiting Intimal Hyperplasia in Rat Models

**DOI:** 10.5935/abc.20180247

**Published:** 2019-01

**Authors:** Tianshu-Chu, Congrong-Gao, Zhiwei-zhao, Fei-Ling, Ayu-Sun, Yuanbiao-Zheng, Jing-Cao, Ge Jianjun

**Affiliations:** Anhui Medical University, Hefei - China

**Keywords:** Myocardial Revascularization/surgery, Cyanocrylates, Sirolimus, Hyperplasia, Graft Occlusion, Vascular, Vascular Patency, Rats

## Abstract

**Background:**

Vein graft restenosis has an adverse impact on bridge vessel circulation and patient prognosis after coronary artery bypass grafting.

**Objectives:**

We used the extravascular supporter α-cyanoacrylate (α-CA), the local application rapamycin/sirolimus (RPM), and a combination of the two (α-CA-RPM) in rat models of autogenous vein graft to stimulate vein graft change. The aim of our study was to observe the effect of α-CA, RPM, and α-CA-RPM on vein hyperplasia.

**Methods:**

Fifty healthy Sprague Dawley (SD) rats were randomized into the following 5 groups: sham, control, α-CA, RPM, and α-CA-RPM. Operating procedure as subsequently described was used to build models of grafted rat jugular vein on carotid artery on one side. The level of endothelin-1 (ET-1) was determined by enzyme-linked immunosorbent assay (ELISA). Grafted veins were observed via naked eye 4 weeks later; fresh veins were observed via microscope and image-processing software in hematoxylin-eosin (HE) staining and immunohistochemistry after having been fixed and stored” (i.e. First they were fixed and stored, and second they were observed); α-Smooth Muscle Actin (αSMA) and von Willebrand factor (vWF) were measured with reverse transcription-polymerase chain reaction (RT-PCR). Comparisons were made with single-factor analysis of variance and Fisher’s least significant difference test, with p < 0.05 considered significant.

**Results:**

We found that intimal thickness of the α-CA, RPM, and α-CA-RPM groups was lower than that of the control group (p < 0.01), and the thickness of the α-CA-RPM group was notably lower than that of the α-CA and RPM groups (p < 0.05).

**Conclusion:**

RPM combined with α-CA contributes to inhibiting intimal hyperplasia in rat models and is more effective for vascular patency than individual use of either α-CA or RPM.

## Introduction

Coronary artery bypass grafting (CABG) is one of the main therapies for coronary heart disease. However, 40% of bridge vessels are totally obstructed and 30% of bridge vessel blood flow is reduced after CABG, which seriously affects patient survival and prognosis.^1^^,^^2^ Mechanisms of restenosis include thrombosis, intimal hyperplasia, and atherosclerosis. Immigration of endothelial cells and vascular smooth muscle cells is vital for intimal hyperplasia, which is the main cause of restenosis.^3^

Although drugs for inhibiting cytokinin and cell cycle regulation contribute to inhibiting intimal hyperplasia, the systemic side effects are harmful for patients. Therefore, local application is very important. Rapamycin (sirolimus) is widely used for anti-rejection after transplant operations, and drug-eluting stents are widely used in coronary arteries. Researchers have found that applying rapamycin to grafted veins is effective in inhibiting intimal hyperplasia by inhibiting proliferation and promoting apoptosis of smooth muscle cells.^4^

In 1963, Parsonnet et al. observed that perivenous supporters were effective for vascular patency.^5^ Subsequently, basic and clinical researchers found that perivenous supporters could enhance patency rates by reducing intimal hyperplasia in grafted veins. α-CA, which is liquid at room temperature, is harmless to the human body. Degradation time is 1-3 months, depending on the dosage. α-CA is used in surgery for bleeding closure and wound binding.^6^

α-CA and RPM are usually used as perivenous supporters and local applications, respectively. We innovatively investigated the pathophysiological process of neointima hyperplasia in grafted veins after CABG via rat models of autogenous vein graft. We are interested in finding new methods to inhibit intimal hyperplasia.

## Methods

### Reagent and method

α-CA (99% n-octyl-α-cyanoacrylate + n-butyl-α-cyanoacrylate) was purchased from Beijing Fuaile Science and Technology Development Co. (Beijing, China). RPM was purchased from Selleck Company. We dissolved 8 mg of RPM in 1 ml of α-CA (taken by pipette) in a sterile EP tube. A magnetic stirrer was then used to mix them to α-CA-RPM of 8 mg/ml, stored in a refrigerator between 2-8°C. RPM was hydrosolvent, prepared by the same method.^7^

### Models and groups

Fifty SD rats (provided by Anhui Lab Animal Research Center and identified by the medical ethics committee of Anhui Medical University), male and female, aged 10-12 weeks, weighing 220-280 g, were randomized (completely randomized design) into 5 groups, each group containing 10 rats, and fed for 4 weeks after operation. Operating procedure and sample size were determined according to pilot experiments and previous studies, as subsequently described.

Operating procedure: an intraperitoneal injection of 10% chloralic hydras was used to anaesthetize rats. Heparin (700 IU/Kg) was injected through the caudal vein to induce heparinization. A vertical incision of approximately 1 cm was made in the middle of the neck (deflected to the operation side), and veins were dissociated on one side. Epitheca of 1-2 mm were taken from 20G red arterial puncture needle (BD Company), used as cannula. The carotid artery was isolated until the branches. Then two suture traction lines and hemoclips were placed at both ends of the artery to block blood flow. The middle of the artery was isolated and turned carefully to 1-1.2 mm above the cannula. A 6/0 silk suture was used to knot and fix in order to isolate the vein from arteries; we were then able to open vascular clamps. The incision was sutured after we verified that the pulse of the grafted vein was normal and there was no bleeding. We checked rats’ vital status and incisions every day. We maintained the environment cool, changed their bedding regularly, and gave them sufficient fodder and water. Three days after the operation, 400,000 IU penicillin were delivered via intramuscular injection to every rat on a daily basis.

Sham group: we merely simulated the operation process. Jugular veins were dissociated and collateral vessels were ligatured, without dividing or transplanting; Control group: jugular arteriovenous graft on the same side; α-CA group: jugular arteriovenous graft and application of α-CA glue to grafted veins; RPM group: jugular arteriovenous graft and application of RPM to grafted veins; α-CA-RPM group: jugular arteriovenous graft on the same side and application of α-CA-RPM to grafted veins.

### Collection of samples

Blood samples were taken preoperatively at 0 h and postoperatively at 12 h, 36 h, and 4 weeks after operation. Serum was collected by centrifugation and stored at -80°C until cytokine analysis. Four weeks later, we collected each group’s vein sample. Fully anaesthetized rats were fixed on the operating table, heparinized as previously described and operated in the same way through the same path. We observed grafted veins’ shapes and circulation and ligatured and isolated vessels at both ends of cannulas; we then removed intact and fresh veins and washed lumens fully with normal saline. Samples with HE staining and immunohistochemistry were placed in microtubes full of paraformaldehyde. Samples with RT-PCR were placed in microtubes full of RNA-EZ regents and then kept in the fridge at -80°C. Rats were euthanized by cervical dislocation method and handled properly.

### Enzyme-linked immunosorbent assay for ET-1

ET-1 was determined by ELISA Kits (R&D, USA) using 50 *µ*l of serum for the assay. Three measurements were performed for each blood sample. The ELISA plate was read at 450 nm in a plate reader.

### Histological examination of graft tissue

Immersed in formalin, grafted veins were cut into 4 mm sections. Hematoxylin-eosin (HE) staining was subsequently performed using a hematoxylin and eosin staining kit (Beyotime Biotechnology, Shang Hai, China). Olympus microscope image acquisition system was used to collect section images (×100 objective lens) and measure intima thickness. Two independent researchers performed the measurements and data analysis. Sections were selected randomly from grafted and non-grafted veins; we then measured 16 points’ thickness and calculated the mean. Three sections were selected and measured from every rat. We then calculated intima thickness.

### Determination of proliferation index

Tissue sections were incubated with the immunohistochemistry analysis kit for proliferating cell nuclear antigen (PCNA) (Santa Cruz Biotechnology, Dallas, TX) at 4°C overnight. After washing with phosphate-buffered saline (PBS) (DAKO, Glostrup, Denmark) and incubating with the secondary antibody, color was developed using the DAB system. The tissue sections were dehydrated and installed on slides. All images (×200 objective lens) were captured by Olympus microscope image acquisition system and SPOT Digital Camera (Diagnostic Instruments, Sterling Heights, MI). PCNA-positive cells were counted in the intima. A total of 10 observation views were used to calculate the average percentage of PCNA-positive cells for each rat.

### RT-PCR

Total RNA of the vessel tissues was isolated by the TRIzol Kit (Life Technology, USA). The RNA was reverse-transcripted to cDNA using the RNA reverse transcription kit (Promega, USA). 2 *µ*g total RNA and 1 *µ*l of random primer were denatured at 70°C for 10 min and annealed at 4°C for 10 min, and then 2 *µ*l of 10× buffer, 2 *µ*l of MgCl_2_ (20.8 mol/l) and 1 *µ*l of reverse transcriptase were added to the reaction system. Double distilled water (ddH_2_0) was added to bring the volume to 20 *µ*l. The condition for cDNA synthesis was 37°C for 1 h and 4°C for 10 min. The PCR also contained 10 *µ*l 2× SYBR Mixture (Takara, Japan), 7 *µ*l ddH_2_0 and 1 *µ*l forward and 1 *µ*l reverse primers. The PCR conditions were 95°C for 5 min, 95°C for 15 s, 60°C for 60 s, and 40 cycles. The sequences of the primers used for RT-PCR were as follows: Forward, 5'-CATCTCCGTGGTCCTGAAGT-3' and reverse, 5'-GGCAAGGGAAACGTCTAGTG-3' for von Willebrand factor; forward, 5'-CAGAGTCCAGCACAATACCAG-3' and reverse, 5'-GACCCAGATTATGTTTGAGACC for α-Smooth Muscle Actin ; and forward, 5'-ACATGAATGACCTCGTCTCTGA-3' and reverse, 5'-CCTCTTCTTCTGCCTCCTCTCC-3' for GAPDH. The instrument for quantitative real-time PCR was purchased from ABI (USA).

### Statistical analysis

All data were analyzed using statistical analysis software SPSS 17.0. Data are presented as mean ± standard deviation. Because data showed a normal distribution, comparison among multiple groups was analyzed by single-factor analysis of variance (ANOVA) and comparison between two groups was conducted by Fisher's least significant difference (LSD) test. A value of p < 0.05 was considered statistically significant.

## Results

### Rats survived well 4 weeks after operation

**Figure 1:**
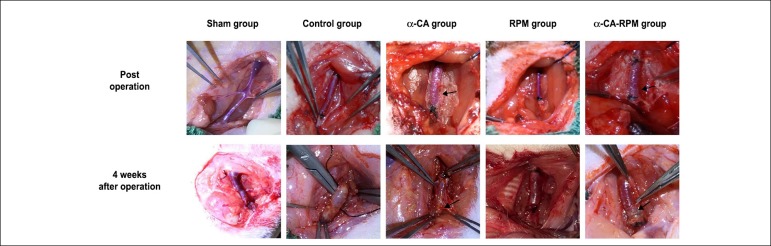
Rats survived well 4 weeks after operation. Operating procedure as previously described was used to build models of grafted rat jugular vein on carotid artery on one side. Post-operation, the transplanted veins are well filled and the blood vessels beat well, and the glue was spread evenly over the surface of the veins in the α-CA and α-CA-RPM groups (arrow). Four weeks after operation, veins in the sham group slightly expanded; the control group had new granulation tissue, thickening tubes, edema and light stiffness; the α-CA group had few fresh tissues which were easily separated, with no obvious expansion and clear boundary from the surrounding, and the glue was not fully degraded (arrow); the RPM group had clear boundaries from the surrounding tissue, and they were fresh and no obvious expansion. The general form of α-CA-RPM group was similar to α-CA group.

Operating procedure as previously described was used to build models of grafted rat jugular vein on carotid artery on one side. Post-operation, the transplanted veins were well filled and the blood vessels beat well; the glue was spread evenly over the surface of the veins in the α-CA and α-CA-RPM group. Rats’ vital status and incisions were checked every day. Subsequently, we found that one rat in the RPM group and one rat in the α-CA group had died of low temperature 2 weeks after operation and the other rats survived and recovered well with strong pulse in grafted veins. The rats were euthanized 4 weeks after surgery; notably, there were only 2 rats who presented venous occlusion, one in the α-CA group and one the RPM group. Correspondingly, blood flow in other grafted veins was patent. Veins in the sham group slightly expanded. What is more, veins in the control group had new granulation tissue, thickened tubes, edema, and light stiffness; however, veins in the α-CA, RPM, and α-CA-RPM groups had few fresh tissues which were easily separated, with no obvious expansion and clear boundary from the surrounding, and the glue was not fully degraded (Figure 1).

### α-CA-RPM reduced intimal thickening of the vein graft

**Figure 2:**
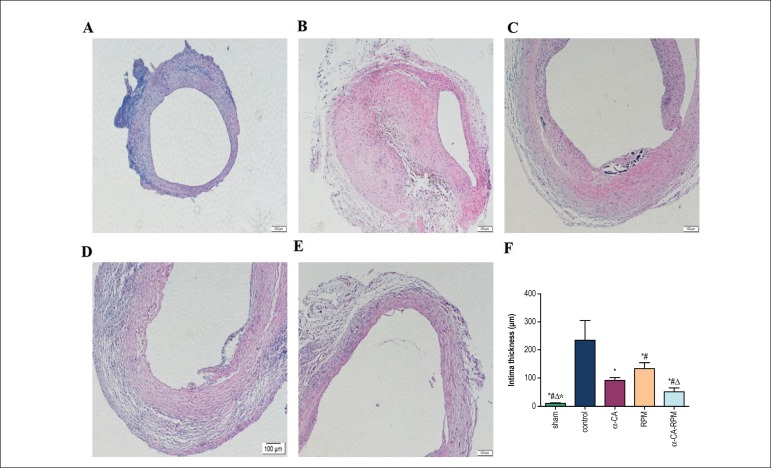
α-CA-RPM reduced intimal thickening of the vein graft. The vessel tissue was harvested 4 weeks after the operation, fixed in formalin, sliced to 4 *µ*m tissue sections and stained with HE. Images (×100 objective lens) were collected and analyzed by Olympus micro-imaging system. The rats were divided into 5 group: Sham group (A), control group (B), α-cyanoacrylate group (C), Rapamycin group (D) and α-CA-RPM group (E). (F) Represented the statistical graph of each group’s intima thickness. The intima of control group was dramatically thicker than that of α-CA, RPM and α-CA-RPM groups; the difference was statistically significant (91.3 ± 3.9, 133.6 ± 8.0, 50.6 ± 5.4 vs. 233.6 ± 29.1 *µ*m, p < 0.01); the intima of the RPM group was thicker than that of α-CA group (133.6 ± 8.0 vs. 91.3 ± 3.9 *µ*m, p < 0.05); the intima of α-CA group and RPM group was thicker than that of α-CA-RPM group (50.6 ± 5.4 vs. 91.3 ± 3.9 *µ*m, 133.6 ± 8.0 *µ*m, p < 0.05). * The control group had obvious difference with other groups, p < 0.05. ^#^ The α-cyanoacrylate group had obvious difference with other groups, p < 0.05. ^△^ The rapamycin group had obvious difference with other groups, p < 0.05. ^☆^ The α-CA-RPM group had obvious difference with other groups, p < 0.05.

**Figure 3:**
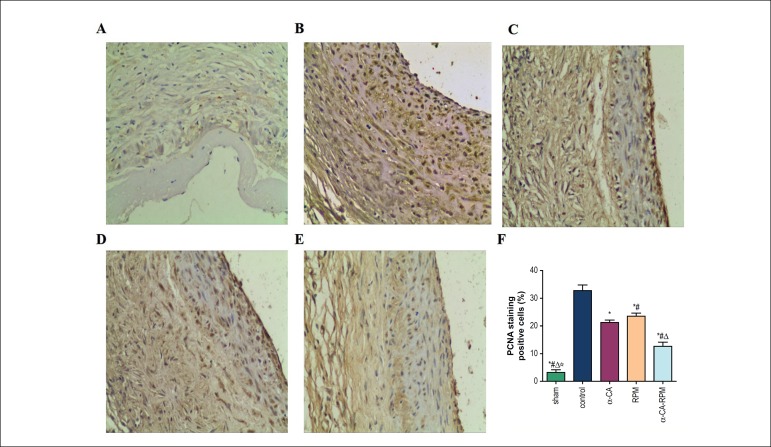
α-CA-RPM decreased the proliferating index of vein graft. The vessel tissue was harvested 4 weeks after the operation, fixed with formalin, sliced to 4 *µ*m tissue sections and stained with the primary antibody anti-PCNA. Images (×200 objective lens) were collected and analyzed by Olympus micro-imaging system. Likewise, the rats were also divided into 5 group: Sham group (A), control group (B), α-cyanoacrylate group (C), Rapamycin group (D) and α-CA-RPM group (E). (F) Represented the statistical graph of each group’s PCNA proliferation index. * The control group had obvious difference with other groups, p < 0.01. ^#^ The α-cyanoacrylate group had obvious difference with other groups, p < 0.01. ^△^ The rapamycin group had obvious difference with other groups, p < 0.01. ^☆^ The α-CA-RPM group had obvious difference with other groups, p < 0.01.

In order to observe what impacts each group’s intervention had on intimal hyperplasia, grafted veins were stained with HE 4 weeks after surgery. Afterwards, we used computer image analysis system to analyze intimal hyperplasia. This showed that the intima of the control group was strikingly thicker than that of α-CA group, RPM group, and α-CA-RPM group; the difference was statistically significant (91.3 ± 3.9, 133.6 ± 8.0, 50.6 ± 5.4 vs. 233.6 ± 29.1 *µ*m, p < 0.01; Figure 2B, C, D, E and F); the intima of the RPM group was thicker than that of α-CA group; the difference was statistically significant (133.6 ± 8.0 vs. 91.3 ± 3.9 *µ*m, p < 0.05; Figure 2C, D and F); the intima of α-CA group and RPM groups was thicker than that of α-CA-RPM group; the difference was statistically significant (50.6 ± 5.4 vs. 91.3 ± 3.9 *µ*m, 133.6 ± 8.0 *µ*m, p < 0.05; Figure 2 C, D, E and F). What is more, as shown in Figure 3, our results from immunohistochemical staining of PCNA demonstrate that the control, α-CA, RPM, and α-CA-RPM groups had a significantly higher proliferating index than the sham group (p < 0.01; Figure 3A, B, C, D, E and F), and the percentage of PCNA-positive cells in the α-CA, RPM, and α-CA-RPM groups was significantly less than in the control group (p < 0.01; Figure 3B, C, D, E and F). Moreover, it is worth noting that the proliferating index in the α-CA-RPM group was markedly less than in the α-CA or RPM group (p < 0.01; Figure 3C, D, E and F). Taken together, our results strongly demonstrate that α-CA, RPM, and α-CA-RPM inhibit intimal hyperplasia in vein grafts, and the effect of α-CA-RPM is stronger than that of α-CA or RPM.

### α-CA-RPM diminished intimal hyperplasia and inflammatory responses

**Figure 4:**
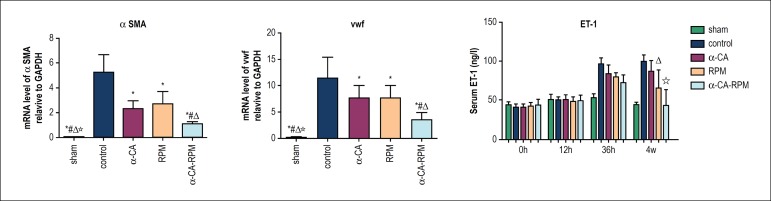
α-CA-RPM diminished the expression of αSMA and vWF and inflammatory responses. Four weeks after the surgery, RT-PCR was used to detect αSMA and vWF in grafted veins. (A) Value of αSMA in α-CA, RPM, and α-CA-RPM groups was much lower than in the control group, as detected by RT-PCR. Value of αSMA in α-CA-RPM group was lower than that of α-CA group and RPM group (p < 0.01). (B) Similar results were found in the value of vWF in α-CA-RPM group (p < 0.01). (C) The serum levels of ET-1 are shown for each group at different times. The level of ET-1 in the control group was significantly higher than that in the α-CA, RPM and α-CA-RPM groups 36 hours and 4 weeks after operation (96.1 ± 7.9 ng/l vs. 84.0 ± 10.9 ng/l, 79.5 ± 5.7 ng/l and 72.7 ± 9.9 ng/l; 99.7 ± 7.7 ng/l vs. 87.1 ± 13.3 ng/l, 65.4 ± 23.4 ng/l and 43.7 ± 20.1 ng/l; p < 0.05, respectively). Additionally, at 4 weeks after surgery, the level of ET-1 of the α-CA-RPM group was significantly lower than that of the α-CA, RPM, and control groups, (43.7 ± 20.1 ng/l vs. 87.1 ± 13.3 ng/l, 65.4 ± 23.4 ng/l and 99.5 ± 7.7 ng/l; p < 0.05, respectively). * The control group had obvious difference with other groups, p < 0.01. ^#^ The α-cyanoacrylate group had obvious difference with sham group, control group and α-CA-RPM group, p < 0.01. ^△^ The rapamycin group had obvious difference with sham group, control group and α-CA-RPM groups, p < 0.01. ^☆^ The α-CA-RPM group had obvious difference with other groups, p < 0.01.

In order to further study the mechanism through which the three intervening methods prevent intimal hyperplasia, we examined the value of αSMA and vWF in grafted veins 4 weeks after surgery. The αSMA values in the α-CA, RPM, and α-CA-RPM groups were much lower than in the control group, as detected by RT-PCR (p < 0.01; Figure 4A). The αSMA values in the α-CA-RPM group were lower than in the α-CA and RPM groups (p < 0.01; Figure 4A). Similar results were found in the value of vWF in α-CA-RPM group (p < 0.01; Figure 4B). This result verified that α-CA, RPM, and α-CA-RPM inhibition might reduce intimal hyperplasia by blocking αSMA and vWF over-expression.

In order to investigate the effect of α-CA-RPM on inflammatory responses, we performed ELISA assays to examine serum levels of ET-1. We found the ET-1 level of the control, α-CA, RPM, and α-CA-RPM groups gradually increased 36 hours after operation; those of the control, α-CA, and RPM groups was still high 4 weeks after operation and that of the α-CA-RPM group had basically returned to normal. The ET-1 level in the control group was significantly higher than that of the α-CA, RPM, and α-CA-RPM groups 36 hours and 4 weeks after operation (96.1 ± 7.9 ng/l vs. 84.0 ± 10.9 ng/l, 79.5 ± 5.7 ng/l, and 72.7 ± 9.9 ng/l; 99.7 ± 7.7 ng/l vs. 87.1 ± 13.3 ng/l, 65.4 ± 23.4 ng/l, and 43.7 ± 20.1 ng/l; p < 0.05, respectively). Additionally, at 4 weeks after surgery, the ET-1 level of the α-CA-RPM group was significantly lower than in the α-CA, RPM, and control groups (43.7 ± 20.1 ng/l vs. 87.1 ± 13.3 ng/l, 65.4 ± 23.4 ng/l, and 99.5 ± 7.7 ng/l; p < 0.05, respectively) (Figure 4C). These findings indicate that α-CA, RPM, and α-CA-RPM seem to reduce inflammatory responses and that α-CA-RPM is more effective.

## Discussion

The main finding of our study is that the application of α-CA, RPM, or α-CA-RPM can improve the patency of the vein graft in rat models by inhibiting intimal hyperplasia.^8^ More importantly, the use of RPM combined with α-CA is more effective than either α-CA or RPM alone.

The complicated remodeling process of vessels leads to restenosis of vein grafts, but the exact mechanism is not explicit. Studies have shown that restenosis is related to dysfunction of intima endothelial cells, proliferation, immigration of vascular smooth muscle cells, adventitial fibroblasts, inflammatory reaction, shear force, and hemodynamic changes.^9^^,^^10^ The pathological process of restenosis of bridge vessel may include early thrombosis, intimal hyperplasia, and atherosclerosis; intimal hyperplasia is the most important reason. When separating, ligaturing, dividing, transplanting, and revascularizing bridge vessels, factors such as aggregation of platelets and neutrophils, release of cytokine and chemokine, activation of transduction pathway, and enzymatic reaction may prompt hyperplasia of vascular smooth muscle cells and accumulation of endothelial cells. All these factors are expected to result in intimal hyperplasia, and restenosis of grafted vessels follows.^11^^,^^12^

In 1963, Parsonnet and colleagues first pointed out that extravascular supporters could enhance the patency rate of grafted veins.^5^ Several foundational and clinical tests have proved that extravascular supporters could inhibit intimal hyperplasia and enhance patency rate. Four extravascular supporters have been widely used in foundational and clinical tests, i.e., nitinol extravascular stent, polymeric extravascular stent, fibrin glue extravascular supporter, and α-CA. It is acknowledged that α-CA not only can prevent post-transplantation vessels from expansion, but also can prompt vascular smooth muscle cells’ migration to vascular outer membrane.^13^^,^^14^ Stimulated by α-CA, many neutrophils and monocytes aggregated to adventitia, especially mononuclear phagocytes which can release amounts of chemotactic factors to attract vascular smooth muscle cells and fibroblast immigration and colonization.^15^ A series of changes mentioned above will activate a range of antiatherosclerotic factors: NO, PGI2, cAMP and cGMP. They can also decrease intimal cholesterol and inhibit pro-atherosclerotic factors.^16^ Outcomes in our experiment revealed that veins in the α-CA group had few fresh tissues and were easy to separate and had clear boundary from the surrounding tissues 4 weeks after the operation. The glue was not fully degraded and the intima of α-CA group was thinner than in the control group. Additionally, the percentage of PCNA-positive cells was significantly less than in the control group. Most importantly, α-CA as the extravascular supporter was able to inhibit intimal hyperplasia and enhance the patency rate.

The proliferation, immigration, and secretion of vascular smooth muscle cells are key to intimal hyperplasia, which contribute to restenosis of vein grafts. Although certain drugs are effective for inhibiting intimal hyperplasia by inhibiting cytokinin and regulating cell cycle, severe toxic reactions and side effects limit their extensive use, as a consequence of which local application becomes particularly significant. RPM, colchicine, and other drugs are used locally on grafted veins. After anastomosis, these drugs are smeared evenly on grafted veins. RPM can accelerate vascular smooth muscle cells’ apoptosis by inhibiting the transformation of cells from G1 to S phase, thus suppressing vascular smooth muscle cells’ proliferation and immigration. Additionally, RPM protects endothelial cell function and reduces the release of vasoactive peptide when endothelial cells get injured.^17^^-^^19^ Furthermore, RPM can also inhibit the differentiation, proliferation, and immigration of endothelial progenitor cells (EPC) and reduce NOS-mRNA expression in EPC.^20^^,^^21^ Our results verified that veins in the RPM group had clear boundaries from the surrounding tissue; they were also fresh and clearly not expanded. Moreover, the intima of the RPM group was thinner than the control group’s and the percentage of PCNA-positive cells was remarkably lower than in the control group. In summary, RPM may inhibit intimal hyperplasia and enhance patency rate.

This study aimed to experiment the combination of an extravascular supporter and a local drug application. We chose α-CA as the extravascular supporter, RPM as the local application, and α-CA-RPM as the combination. α-CA-RPM was used in rat models of autogenous vein graft to stimulate grafted veins’ pathophysiological process after CABG. Interestingly, we found the percentage of PCNA-positive cells in the α-CA-RPM group was markedly less than in the control, α-CA, and RPM groups, which indicated that α-CA-RPM was more effective in inhibiting intimal hyperplasia than either α-CA or RPM separately. We concluded that α-CA-RPM can combine the effectiveness of extravascular supporters and local drugs and thus better inhibit intimal hyperplasia. Meanwhile, α-CA is an ideal carrier for the formulation of long-term control drug release which surrounds the vein graft tightly so that RPM will be released slowly and no RPM will be wasted.

The endothelin-1 (ET-1) has been implicated in the pathogenesis of restenosis and vascular hypertrophy via enhancing aggregation of platelets and neutrophils, release of cytokine and chemokine, accumulation of endothelial cells, and promotiong of vascular smooth muscle cell migration towards the intimal layer.^22^ Our results indicate that α-CA, RPM, and α-CA-RPM can stabilize endothelial cell function and diminish the release of ET-1 to inhibit intimal hyperplasia. An endothelin A/B receptor antagonist contributed to reduction of intimal hyperplasia in an organ culture of human saphenous veins and prevented neointimal development of coronary angioplasty in pigs, which is in accordance with our experiment.^23^^,^^24^ αSMA is the specific protein of vascular smooth muscle cells and the expression of αSMA can reflect the hyperplasia of vascular smooth muscle cells. In our experiment, we examined the values of αSMA in grafted veins with RT-PCR and found that the values in the α-CA, RPM, and α-CA-RPM groups was lower than in the control group. Notably, the value of the α-CA-RPM group was lower than that of the α-CA and RPM groups. A study in which the αSMA component of vascular progenitor cells correlated with the coronary artery Gensini score also made the same point.^25^ An experiment in a swine model of arteriovenous bypass grafting also provided tangible evidence to support this point of view.^26^

The vWF is a glycoprotein encoded by the short arm of chromosome 12 and can be combined with collagen fibers and platelets; it is closely related to a range of cardiovascular diseases such as atherosclerosis, acute coronary syndrome, and atrial fibrillation.^27^ vWF directly stimulates vascular smooth muscle cell proliferation, resulting in a direct dose-response effect. It also accelerates intimal hyperplasia in intact endothelium without platelet activation or platelet-derived growth factor release.^28^ Likewise, we found the vWF values of the α-CA, RPM, and α-CA-RPM groups was lower than that of the control group, and the α-CA-RPM group was lower than the α-CA or RPM groups. Our results in rats have been supported by experiments in other animals, such as an efficacy study in dogs and intimal hyperplasia of rabbit carotid arteries.^29^^,^^30^ These results demonstrate that α-CA, RPM, and α-CA-RPM might reduce intimal hyperplasia by blocking ET-1, αSMA, and vWF overexpression.

Our results show that rapamycin combined with α-cyanoacrylate contributes to inhibiting intimal hyperplasia and is more effective for vascular patency than individual use of either α-CA or RPM in rat models 4 weeks after operation. The long-term effects of α-CA-RPM on vein graft remodeling are still unclear. Our team will conduct further research on intimal hyperplasia pathophysiological processes in pigs after CABG and the impacts of related interventions on grafted veins.

## Conclusion

Our results confirmed that α-CA-RPM contributes to inhibiting intimal hyperplasia and is more effective for vascular patency than individual use either α-CA or RPM in rat models of artery bypass grafting. The positive effects appear to be associated with decreased intimal thickening, reduced cell proliferation in the vein graft, and decreased inflammatory responses. Although the shor-term effects of α-CA-RPM seem promising, the long-term effects and clinical significance of α-CA-RPM in CABG need to be studied in the future.
